# Perceptions from people with physical disabilities about accessibility and social conditions: interventions for rehabilitation nursing

**DOI:** 10.1590/0034-7167-2024-0005

**Published:** 2024-11-22

**Authors:** Rute Salomé da Silva Pereira, Salomé Sobral Sousa, Maria Manuela Martins, Wiliam César Alves Machado, Soraia Dornelles Schoeller

**Affiliations:** I Universidade do Porto. Porto, Portugal; II Universidade do Porto, Centro de Investigação em Tecnologias e Serviços de Saúde e Rede de investigação em Saúde. Porto, Portugal; III Escola de Enfermagem Alfredo Pinto. Urca, Rio de Janeiro, Brazil; IV Universidade Federal de Santa Catarina. Florianópolis, Santa Catarina, Brazil

**Keywords:** People with Disabilities, Rehabilitation Nursing, Social Conditions, Architectural Accessibility, Sustainable Development Goals, Pessoas com Deficiência, Enfermagem em Reabilitação, Condições Sociais, Acessibilidade Arquitetónica, Objetivos de Desenvolvimento Sustentável, Personas con Discapacidad, Enfermería en Rehabilitación, Condiciones Sociales, Accesibilidad Arquitectónica, Desarrollo Sostenible

## Abstract

**Objectives:**

to analyze the experiences of people with acquired physical disabilities regarding accessibility and social conditions; to identify nurse-led rehabilitation interventions for accessibility and social conditions; to determine nursing-sensitive indicators to improve accessibility and social conditions.

**Methods:**

a descriptive-exploratory qualitative study employed semi-structured interviews with people with acquired physical disabilities through purposive snowball sampling to address all objectives. Data analysis followed Bardin’s content analysis principles. Furthermore, objectives 2 and 3 were achieved through a reflective theoretical approach.

**Results:**

the 27 participants reported accessibility challenges, impacting activities of living and social conditions. This influences rehabilitation nursing, leading to three intervention fields: Assess the ability to perform activities of living and influencing factors; Develop and implement training to perform activities of living; Promote mobility, accessibility, and social participation.

**Final Considerations:**

based on participants experiences, we identified nurse-led rehabilitation interventions to promote accessibility and social conditions.

## RESUMO

**Objetivos:**

analisar experiências de pessoas com deficiência física adquirida quanto à acessibilidade e condições sociais; identificar intervenções de reabilitação lideradas por enfermeiros para acessibilidade e condições sociais; determinar indicadores sensíveis à enfermagem para melhorar acessibilidade e condições sociais.

**Métodos:**

estudo qualitativo descritivo-exploratório empregou entrevistas semiestruturadas com pessoas com deficiência física adquirida por meio de amostragem proposital em bola de neve para atender a todos os objetivos. Análise dos dados seguiu os princípios da análise de conteúdo de Bardin. Objetivos 2 e 3 foram alcançados através de abordagem teórica reflexiva.

**Resultados:**

os 27 participantes relataram desafios de acessibilidade, impactando atividades de vida diária e condições sociais. Isto influencia a enfermagem de reabilitação, conduzindo a três campos de intervenção: Avaliar a capacidade de realizar atividades diárias e fatores influenciadores; Desenvolver e implementar treinamento para realização de atividades diárias; Promover mobilidade, acessibilidade e participação social.

**Considerações Finais:**

com base nas experiências dos participantes, identificamos intervenções de reabilitação lideradas por enfermeiros para promover acessibilidade e condições sociais.

## RESUMEN

**Objetivos:**

analizar las experiencias de personas con discapacidad física adquirida en cuanto a accesibilidad y condiciones sociales; identificar intervenciones de rehabilitación dirigidas por enfermeras para la accesibilidad y condiciones sociales; determinar indicadores sensibles a la enfermería para mejorar la accesibilidad y condiciones sociales.

**Métodos:**

estudio cualitativo descriptivo-exploratorio utilizó entrevistas semiestructuradas con personas con discapacidad física adquirida mediante muestreo intencionado en bola de nieve para cumplir con todos los objetivos. El análisis de datos siguió los principios del análisis de contenido de Bardin. Los objetivos 2 y 3 se lograron mediante un enfoque teórico reflexivo.

**Resultados:**

los 27 participantes informaron desafíos de accesibilidad, que impactan las actividades de la vida diaria y las condiciones sociales. Esto influye en la enfermería de rehabilitación, dando lugar a tres campos de intervención: Evaluar la capacidad para realizar actividades diarias y los factores que influyen; Desarrollar e implementar capacitación para la realización de actividades diarias; Promover la movilidad, la accesibilidad y la participación social.

**Consideraciones Finales:**

a partir de las experiencias de los participantes, identificamos intervenciones de rehabilitación dirigidas por enfermeras para promover la accesibilidad y condiciones sociales.

## INTRODUCTION

Accessibility is a central principle of the Convention on the Rights of Persons with Disabilities that enables persons to live independently and participate in all aspects of life on an equal basis with others^(1)^. It is a right in itself; however, it is also an instrumental right to access other rights such as education, health, or work.

The influence of environmental factors, such as architectural barriers, on people’s lives is undeniable. This impact reflects on the social conditions in which people with acquired physical disability (PwAPD) live and work^(2,3)^. Although disability is not synonymous with poverty and social exclusion, it brings additional costs to households, affecting living conditions^(4-6)^.

Rehabilitation nurses promote health and prevent further disability by managing care for people with acute or chronic health conditions across the lifespan with a clientand family-centered care approach for a successful transition and well-being; in Portugal, it is an established specialty with formal regulation^(7,8)^. Rehabilitation nurses should not only focus on the physical aspects of rehabilitation for PwAPD, but should also provide a holistic care approach where social rehabilitation is essential^(8-11)^. However, there is a lack of specific literature in nursing sciences regarding the role of rehabilitation nurses in enhancing accessibility and social conditions for PwAPD.

These two aspects are essential to achieving the Sustainable Development Goals, and rehabilitation nurses play a pivotal role^(12-14)^. Definitely, rehabilitation nursing contributes significantly to Goal 3: Ensure healthy lives and promote well-being for all at all ages. However, when we talk about accessibility and social conditions, rehabilitation nurses can specifically contribute to achieving Goal 10: Reduce inequality within and among countries by empowering people with disabilities and ensuring equal opportunities through accessibility and social conditions. It also contributes to achieving Goal 11: Make cities and human settlements inclusive, safe, resilient, and sustainable by promoting accessible environments crucial for social inclusion and participation. Nurse-led rehabilitation interventions can help nurses achieve these goals based on the care management process by assessing, planning, implementing, and evaluating, which requires a partnership between nursing theories and the needs expressed and felt by PwAPD.

Nursing theories and conceptual models provide the framework for rehabilitation nursing practice and can guide us to understand the barriers and difficulties experienced by PwAPD. To this end, we choose Roper-Logan-Tierney’s Activities of Living Model, which provides a guide to assess and create an individualized holistic care plan encouraging patient-driven to achieve personal goals, health, and well-being^(15)^.

In this model, the authors emphasize the fact that the environment plays a significant role in a person’s ability to perform daily activities. The environment can either disable or enable full participation, and we can change it to support individuals’ capabilities by addressing both the person and the environment^(2,15-18)^. Once nurses assess the environment that influences all the activities of living, the need to assess architectural accessibility arises.

The Activities of Living Model is based on five main concepts, namely: 12 activities of living (Maintaining a safe environment, Communicating, Breathing, Eating and drinking, Eliminating, Personal cleansing and dressing, Controlling body temperature, Mobilizing, Working and playing, Expressing sexuality, Sleeping, and Dying), lifespan, dependence/independence *continuum*, factors influencing activities of living (biological, psychological, sociocultural, environmental, and political-economic) and individualizing nursing^(15)^.

Another relevant aspect is that rehabilitation nurses implement nursing-sensitive indicators in their clinical practice, enabling objective assessment and an improvement in quality of care. This, in turn, leads to health gains for PwAPD’s well-being^(19)^. The Donabedian model, which is frequently used in healthcare services to assess quality, is established on the triad of structure, process and outcomes^(20)^.

The main question we are trying to answer in this research is: how do PwAPD analyze the social conditions and accessibilities that lead to rehabilitation nursing interventions?

## OBJECTIVES

To analyze the experiences of People with Acquired Physical Disabilities (PwAPD) regarding accessibility and social conditions; to identify nurse-led rehabilitation interventions for accessibility and social conditions based on PwAPD experiences; to determine nursing-sensitive indicators to improve accessibility and social conditions.

## METHODS

### Ethical aspects

The study was conducted in accordance with national and international ethics guidelines and approved by the Research Ethics Committee of ICBAS - School of Medicine and Biomedical Sciences from the *Universidade do Porto*, whose opinion is attached to this submission. From all participants, we obtained written informed consent.

### Theoretical-methodological framework

For this study, we used the theoretical framework of Roper-Logan-Tierney’s Activities of Living Model^(15)^, the Portuguese normative from the Nursing Board Regulation about specific competencies of specialist nurses in rehabilitation nursing^(7)^, and the 2030 Agenda for Sustainable Development^(13)^.

### Study design

We conducted a descriptive-exploratory qualitative study employing semi-structured interviews with PwAPD through a non-probabilistic purposive sampling approach, explicitly implementing snowball sampling to address all three objectives mentioned above. Furthermore, to achieve objectives 2 and 3, we developed a reflective theoretical approach.

We followed the COnsolidated criteria for REporting Qualitative research (COREQ) checklist to improve the quality of reporting qualitative research for the applicable items^(21)^.

### Methodological procedures

#### Study setting and data source

We recruited participants through snowball sampling from various regions across Portugal. Initially, we identified participants through the contact lists of the *Associação Salvador* and the Disability and Human Rights Observatory and via social media channels. Subsequently, we contacted those interested in participating in the study via phone or e-mail. There is no relationship between the participants and the researcher. Inclusion criteria included being a PwAPD for at least one year, living in the community, being 18 years or older and having access to a phone and the internet. We included participants only if they meet the inclusion criteria.

#### Data collection and organization

The COVID-19 pandemic impacted data collection from January to November 2020. The research team developed the interview-scripted guide containing two parts. The first part contains demographic and disability information, and the second part contains a semi-structured script with six nondirective questions: (a) How has the experience of having an acquired physical disability affected your life? What positive and negative aspects have emerged as a result? (b) How is the management and organization of your daily routine handled? (c) What special accommodations does your home have to ensure comfortable living? Have there been any modifications to your home since then? If yes, could you describe the changes made and their reasons? (d) How do you imagine an accessible and inclusive environment, including streets, buildings, and transportation for individuals in situations similar to yours? (e) What role have healthcare professionals played in your life since the onset of your disability? (f) Has there been any discussion with a nurse regarding accessibility and architectural barriers? If not, would it be beneficial to have such a conversation?

We developed a pilot test of the interview guide to ensure that the material and questions were understandable and written in plain language, and we made no alterations. Initially, we reached out to participants to schedule the interviews, and then the main researcher visited participants’ homes and used in-person interviews to collect data. However, after the lockdown due to the COVID-19 pandemic, the interviews were conducted online using Zoom (a web conferencing platform). In both strategies, we used audio records for verbatim transcripts (record or online) after obtaining informed consent. We allowed non-participants, such as family members, to be present if participants wanted. On average, the interviews lasted one hour.

#### Data analysis

The main researcher used verbatim transcript of interview data uploaded to ATLAS.ti software for qualitative data analysis. The two members of the research teams analyzed the transcripts using Bardin’s content analysis method^(22)^. Initially, the lead researcher conducted a pre-analysis of the data and organized the material by conducting preliminary readings, establishing the *corpus* of analysis based on exhaustiveness, representativeness, homogeneity, and relevance criteria.

Following this, in the material exploration phase, we proceeded with coding to identify relevant themes and their respective units of analysis. Subsequently, we organized the material into semantic categories during the categorization phase. During the analysis, the two research teams frequently discussed codes, and themes emerged to find a consensus on coding and themes. We reached data saturation with the 27^th^ interview, since we found the same ideas in participants’ discourses, and no new codes emerged. The research team conducted a reflective theoretical approach from the main categories that emerged to infer rehabilitation nursing interventions and determine nursing-sensitive indicators specifically for those areas.

**Figure 1:**
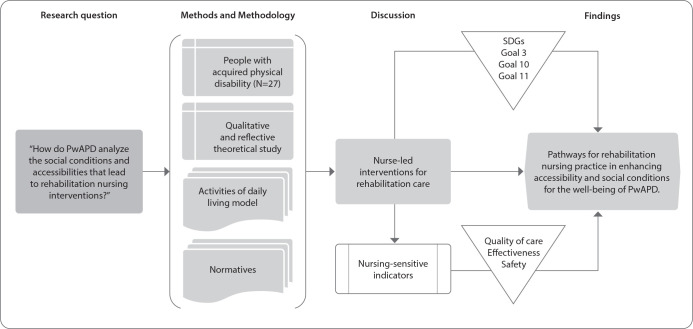
Methodological research path

Figure 1 represents the steps and pathways developed for the study procedures.

## RESULTS

Our sample consisted of 27 PwAPD, 14 male and 13 female. Participants ranged in age from 18 to 71, with a mean age of 40.93 years (SD= 13.37). Almost half of participants are single (48.1%), and education levels and job classifications are diverse.

Half of participants state that their household has between 1 and 2 minimum wages per month, and the primary source of income is mainly other family members (40.8%) and the person with disability (37%). Most participants live in a non-rural area. The main cause leading to acquired physical disability was a spinal cord injury (63%), and most participants used a manual wheelchair (70.4%) (Table 1).

**Table 1:** Sample characteristics (N=27)

Variable	n (%)
Gender	
Female	13 (48.1)
Male	14 (51.9)
Relationship status	
Married	11 (40.8)
Single	13 (48.1)
Divorced	3 (11.1)
Level of education	
Less than high school	7 (25.9)
High school	7 (25.9)
Bachelor’s degree	6 (22.2)
Graduate degree	7 (25.9)
Job classification^$^	
Managers	2 (7.4)
Professionals	7 (25.9)
Technicians and associate professionals	5 (18.5)
Clerical support workers	3 (11.1)
Service and sales workers	1 (3.7)
Craft and related trades workers	1 (3.7)
Not classified/not answered	8 (29.6)
Main source of income	
Family member	11 (40.8)
Own person with disability	10 (37.0)
Retirement income	3 (11.1)
Other	3 (11.1)
Wage^*^ (per month)	
Less than 1 minimum wage	3 (11.1)
1 - 2 minimum wages	15 (55.5)
3 or more minimum wages	8 (29.6)
Not answered	1 (3.7)
Community environment	
Non - rural area	19 (70.4)
Rural area	8 (29.6)
Cause of physical disability	
Spinal cord injury	17 (63.0)
Stroke brain injury	2 (7.4)
Neuromuscular disorder	3 (11.1)
Amputation	2 (7.4)
Other	3 (11.1)
Mobility devices used	
Power wheelchair	6 (22.2)
Manual wheelchair	19 (70.4)
Other devices for walking	2 (7.4)

^*^When the interviews took place in Portugal, the minimum wage was 635€;

$ By International Classification of Occupations (ISCO) - major group classification (https://ilostat.ilo.org/resources/concepts-and-definitions/classification-occupation/).

### Interviews content analysis

Two categories emerged from content analysis: accessibility and social conditions. We further divided the first category about accessibility into nine subcategories: general accessibility; accessibility for social participation; housing accessibility; accessibility in public environment; public building accessibility; transports accessibility; workplace accessibility; information about accessibility; and wheelchair use.

We divided the second category that identifies social conditions into eight subcategories, such as positive and negative aspects of being a person with disability, inclusion, social protection, leisure activities, assistive technology, personal assistant, and working conditions.

### Accessibility category

The accessibility category addresses participants’ views on accessibility conditions, not only inside the house or urban built environment but also at work and transportation, which allows disabled persons to live independently and fully participate in all aspects of life.

The general accessibility subcategory reflects participants’ views on accessibility conditions in general that allow living an active and independent life.

*I think that many times what happens is that there are architectural barriers. It is important to raise awareness, and it is important to address these issues.* (P18)

*Architectural barriers are a significant factor* […] *it enables people to go out into the street, and if they have the conditions to go out into the street, it increases their visibility. Moreover, if their visibility increases, it also makes them play a more active role in society.* (P24)

*The streets should all be smooth, and buildings should have access ramps and elevators inside.* (P4)

In the accessibility for social participation subcategory, participants acknowledged the impact of the environmental conditions or barriers that prevent their involvement in a life situation the same as or even more than individual impairment.

*I already stopped going to some places with other people because there is no access.* (P1)

The housing accessibility subcategory addresses housing conditions, including alterations and modifications that participants made to improve accessibility in home environments to eliminate architectural barriers for independence.

*It seems like we are prisoners in our own home* […] *because ultimately that is exactly how I feel.* (P22)

*We made some modifications in the bathroom, like a roll-in shower.* (P18)

*I needed to make a bathroom and a bedroom accessible for me on the ground floor.* (P9)

In the accessibility in public environment subcategory, participants’ discourses emphasize barriers in urban environments, such as sidewalks.

*Pavement is extremely slippery* […] *on a rainy day when the pavement is wet* […] *the fallen tree leaves are also hazardous* […]. (P22)

The public building accessibility subcategory highlights participants’ experiences with architectural barriers they face when accessing the built environment. It also underlines the fact that it is necessary to design it bearing in mind that PwAPD also desires to access and participate in daily activities.

*Very often, building entrances have small steps that are not considered as a barrier, because for the majority of the people it is just a small step* […]. (P24)

Another subcategory that emerged from the discourses is transports accessibility, in which participants accentuate the barriers in public transportation.

*Here public transportation does not have access to my house.* (P10)

In the workplace accessibility subcategory, participants identify the difficulties they must overcome to access the workplace.

*They made small adaptations, yet some architectural barriers remained, which were resolved almost 15 years after* […]. (P18)

The information about accessibility education subcategory reflects participants’ experiences regarding whether, in their process of acquired disability, they had disability-related accessibility information among providers.

*We always need to find information by ourselves. No one informed us about anything, and Nurses? Never. They would come and ask me for help. They do not have the required training and usually refer to the hospital’s social work offices.* (P27)

*Yes, that would be very helpful in the beginning, particularly for those who were not born with disabilities. It is not easy* […]. (P5)

*It could have been more directed, in the sense of providing support or indicating someone who could provide it!* (P6)

In the wheelchair use category, participants express their need for a mobility device, such as a wheelchair.

*I used to say that my wheelchair does not discourage me, it does not define myself, or deterrents me from doing anything. My wheelchair is a means to an end.* (P27)

### Social conditions category

In the positive aspects of being a person with disability subcategory, we can find what participants identify as positive manifestations or expressions of their experience of being a person with acquired physical disability.

*I met amazing people, with or without disability, people with a wider vision* […]. (P27)

On the other hand, concerning the negative aspects of being a person with a disability subcategory, the discourses express the negative manifestations or expressions of living with a disability.

*Besides living completely dependent on them, the guilt that I carry, and they make me feel is huge* […]. (P11)

*There are a few negative aspects such as accessibility, the indiscreet looks in the street and the typical comments like “you poor thing”* […]. (P17)

Participants emphasized the importance of citizenship education in fostering inclusive societies within the inclusion subcategory.

*More important than being equal is to have a just and equitable society. We cannot all be equal because we are all different. We have different necessities; society must adapt to all kinds of individuals. People are not ready for difference. From the outset, it is important that schools promote a difference acceptance at an early age.* (P27)

In the social protection subcategory, participants mentioned if they received any financial support from the state and acknowledged other types of support that they received. Thirteen participants (P1, P2, P4, P5, P8, P9, P10, P14, P16, P17, P18, P21, P24) received social inclusion benefits.

*I am fortunate to have a family with financial conditions to support me well-timed* […] *these type of assistive devices takes a long time and are difficult to get.* (P24)

The leisure activities subcategory emerged from the discourses because many participants emphasized that they had to stop their regular leisure activities and challenges when travelling.

*I stopped practicing a sport that I enjoyed* […]. (P20)

*Where I live, we do not have adapted sport for persons with disabilities.* (P2)

*We went to Madeira, and travelling by airplane was an adventure. It was the first time I travelled in a wheelchair* […]. (P26)

In the assistive technology subcategory, participants’ discourses address the value these devices have in their daily living. They give examples of which devices they use and the importance of technology in this area.

*I now have autonomy because I can have assistive devices that allow me to compensate for the autonomy that I lost*. (P27)

[…] *transfer board, adjustable bed, a shower bench chair* […]. (P24)

[…] *I can also pull the windows up and down with my phone* […]. (P18)

In the personal assistant subcategory, participants’ discourses highlight the significance of having this kind of assistant for activities of living.

*My assistant dresses me and transfers me to the chair to go to the bathroom to urinate, wash my teeth and face, meanwhile, he prepares and brings me my breakfast* […] *I leave home to get to work.* (P24)

In the working conditions subcategory, some participants are aware of workplace changes after disability.

*I left my work as a cruise cook, now it is difficult to do my job*. (P10)

*I left my job as a practitioner nurse* (P6)

*They ignore my disabilities. They are not interested in knowing whether I have barriers or not if I need any kind of support* (P5)

However, other participants expressed that they could find a new job or maintain their previous job as a nurse with few adaptations.

*I was able to find a job, and I live a normal life.* (P19)

*I just do not do home visiting. I do everything else. I am a family nurse practitioner*. (P26)

## DISCUSSION

The findings reveal PwAPD experiences and the impact of accessibility as well as its implications on their social and living conditions. There is limited evidence from rehabilitation nurses on how nurse-led interventions can enhance accessibility and social conditions for PwAPD. To fill this gap and to support this discussion and interpretations of the data collected, we conducted a reflective theoretical approach to identify nurse-led rehabilitation interventions for accessibility and social conditions based on PwAPD experiences.

We identified three fields for rehabilitation nurse-led interventions for PwAPD: Assess the ability to perform activities of living and the influencing factors; Develop and implement training to perform activities of living; and Promote mobility, accessibility, and social participation.

### Assess the ability to perform activities of living and influencing factors

Rehabilitation nurses already assess the level of functional independence and the client’s ability to manage activities of living, such as maintaining a safe environment, eliminating, personal cleansing and dressing, working and playing. Rehabilitation nurses must assess of the disabled person’s functional ability. However, these abilities are also determined by environmental factors such as architectural barriers^(8,16,23)^. As prior research suggests, participants acknowledged the need to adapt and modify the internal housing environment^(16,24)^.

In this field, rehabilitation nurses are vital in providing information, advocating for, and educating PwAPD and their families, highlighting their capabilities, addressing health needs, and facilitating transitions from clinical settings to home and community environments^(23,25,26)^.

### Develop and implement training to perform activities of living

Maintaining a safe environment and mobilizing are two of the most affected activities of daily living for persons with disability. In our findings, using a wheelchair emerged as a prominent facilitator for maintaining independence in activities of daily living, and prior research indicates the need to incorporate education about wheelchair-related falls in rehabilitation programs^(27,28)^.

Our data indicated that assistive technology plays a significant role in performing activities of daily living more independently, and previous studies reported that assistive technology enables independence for persons with disabilities, which ensures social participation and facilitates finding employment^(29,30)^.

These findings confirm the necessity to implement nursing interventions, such as instructing and training to improve capacity to improve wheelchair skills, prevent falls and use assistive technology.

### Promote mobility, accessibility, and social participation

Accessibility is a significant reported difficulty in our findings. We were not surprised to find that every participant reported problems in accessibility, not only inside their homes but in general, for social participation, in public and built environments, in transport and in the workplace, which concurs with previous studies^(2,3,17,24,31-33)^.

Nurses cannot miss the opportunity to inform, advocate, and educate about architectural barriers and to assess the accessibility of the home and public environment to adapt to home, neighborhood, and other environments in the different contexts of daily living. It reaffirms some interventions rehabilitation nurses have already implemented^(10,32,34,35)^.

Evidence suggests that environmental barriers and lack of suitable jobs are the main reasons to be unemployed^(18,36)^, and returning to work for persons with a disability means that they have to face the lack of sensitivity from employers and attitudinal barriers^(37)^. Our findings support the claim that there is a direct impact on participation in leisure activities and working conditions. Nurses can implement different strategies, which include return-to-work programs, promoting workplace adaptations, being aware of the need to find a different job, improving new skills for returning to work and developing programs for participation in social and leisure activities.

In participants’ discourses, we found positive and negative aspects of being a person with a disability, such as optimism and acceptance of the disability, in contrast with the report of the existence of architectural barriers and the lack of attention to eliminating them, discrimination, indiscreet looks, feeling that they are a burden to family members, which is consistent with previous literature^(26,38)^. By teaching, informing, and advocating, rehabilitation nurses can improve individual competencies that allow them to cope with the transition to living with an acquired disability.

Despite the close relationship between participants’ discourse and the needs they expressed in the educational and training component, the findings of this research lead to the question: do nurses indeed “teach”? We must reflect on this subject because, although rehabilitation nurses already implement interventions in that field, PwAPD feel that nurses do not teach them about their personal needs^(39-41)^. To accomplish this goal, a dynamic approach is required, where dialogue between rehabilitation nurses and PwAPD is the key to an emancipatory process.

One of the participants highlights the importance of inclusion in which, from our point of view, as nurses, we can identify at least four possible ways: (1) develop and implement rehabilitation programs that include transitional care from the hospital to the community setting, comprising the home and community visit; identification and elimination of architectural barriers in the environment^(9,23,42)^; (2) advocate and inform for the rights of persons with disabilities through awareness campaigns such as for the general public and schools^(43,44)^; (3) disability competence in nursing school curriculums^(45,46)^; (4) advocate for local and national policies for persons with disabilities through professional organizations, local governments, and policymakers^(7,8,47)^.

In order to operationalize the three previously identified areas, we presented the following interventions, aligning with the action axis in this domain, namely: “informing” is giving or telling someone facts or information about something; “advocating” is recommending someone or something by argument; “teaching” is giving systematic information to someone about health-related subjects; “instructing” is giving systematic information to someone about how to do something; and “training” is developing skills of somebody or functions of something^(48)^.

**Figure 2:**
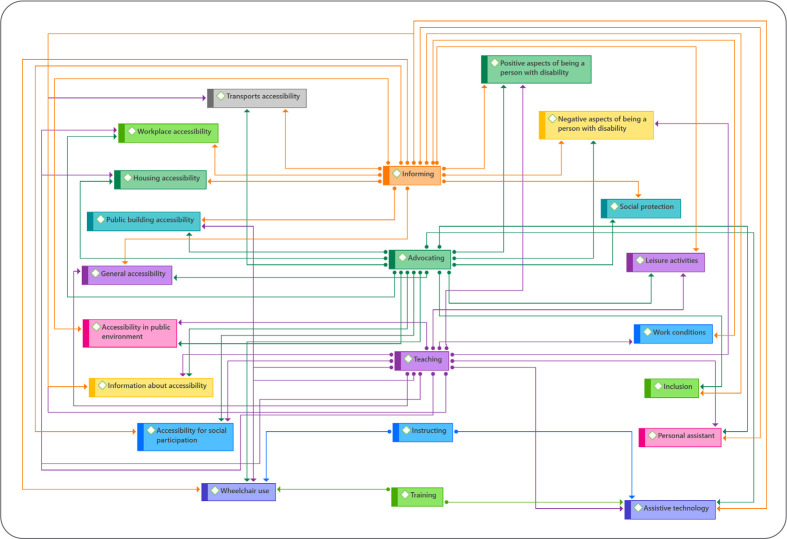
Nurse-led interventions for people with acquired physical disability

In summary, Figure 2 exhibits nurse-led interventions to improve accessibility and social conditions for PwAPD by relating the nursing interventions from the action axis with the emerged categories.

It is essential to determine nursing-sensitive indicators to assess the effectiveness of nursing interventions to enhance accessibility and the social conditions for PwAPD, which allows the establishment of protocols for monitoring results and the quality of nursing care to explain the need to invest in rehabilitation nurses^(20)^.

**Figure 3:**
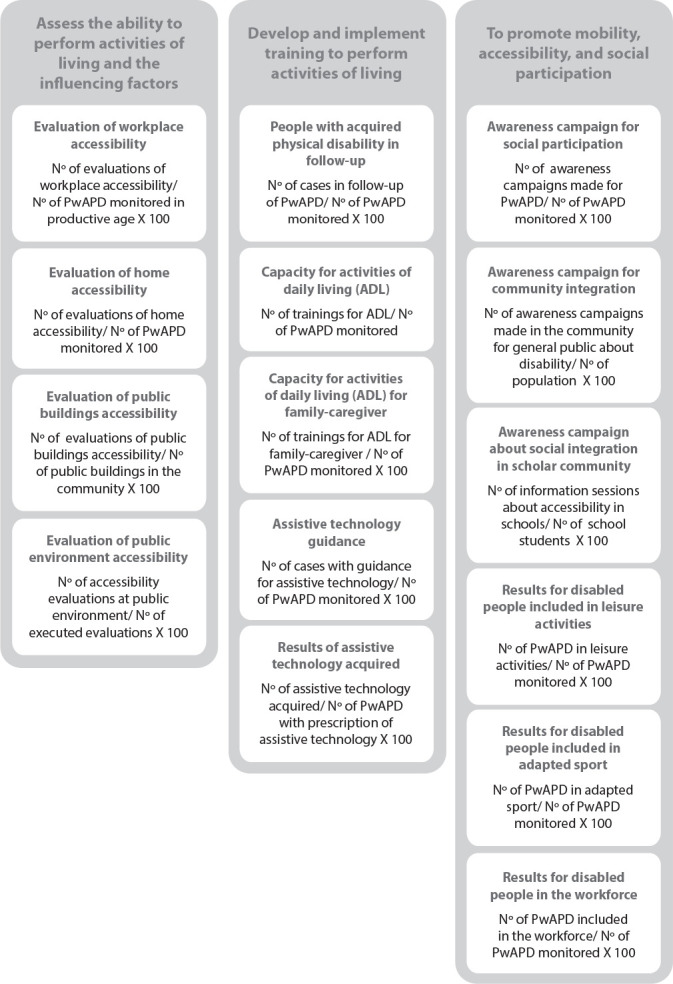
Nursing-sensitive indicators for rehabilitation nursing practice for social conditions and accessibility for people with acquired physical disability

From our analysis of each field for rehabilitation nurse-led interventions presented before, we inferred nursing-sensitive indicators and their operationalization (Figure 3).

### Study limitations

The study limitations include sample size, as the findings represent this specific sample but may not be generalizable to all individuals with disabilities. Participants predominantly live in non-rural areas, which could also impact the results obtained. For future investigations, it is essential to conduct a comparative analysis of accessibility in both rural and urban environments. Additionally, there is a need to advance nursing research focusing on implementing interventions addressing accessibility and social conditions and exploring the application of nursing-sensitive indicators to assess the quality of rehabilitation nursing care.

### Contributions to nursing

This paper highlights nurse-led interventions for rehabilitation nursing care to improve accessibility and social conditions from PwAPD perspective. This reaffirms the need for rehabilitation nurses to assess the physical and built environment in the care management process and implement specific interventions.

We also proposed some nursing-sensitive indicators that can improve quality of care in rehabilitation nursing.

## FINAL CONSIDERATIONS

In this research, the lack of accessibility is a common barrier and difficulty experienced by PwAPD, influencing their activities of living and social conditions. Consequently, this affects their ability to access fundamental rights and liberties. In this regard, our study identified fields for rehabilitation nurse-led interventions for PwAPD, which we believe will contribute to achieving the SDGs.

It is urgent for rehabilitation nurses to take the lead within the multidisciplinary team to develop a rehabilitation care plan and implement holistic rehabilitation programs with specific nursing interventions in partnership with PwAPD based on their lived experiences and personal perspectives, aiming to promote accessibility and social conditions for successful living and well-being.
